# Neuroleptic Malignant Syndrome Caused by a Combination of Carbamazepine and Amitriptyline

**DOI:** 10.1155/2012/183252

**Published:** 2012-07-24

**Authors:** A. Bruce Janati, Naif ALGhasab, Aboubaker Osman

**Affiliations:** ^1^Center For Neurology, Fairfax, VA 22031, USA; ^2^Department of Neuroscience, King Khaled Hospital, 81442 Hail, Saudi Arabia; ^3^Qassim University, Buraydah 51497, Saudi Arabia

## Abstract

A 32-year-old female, with a history of secondarily-generalized convulsive epilepsy, mental retardation, and a psychiatric illness, developed neuroleptic malignant syndrome while receiving carbamazepine and amitriptyline concurrently. We hypothesize that the addition of amitriptyline to carbamazepine caused a decrease in the serum level of carbamazepine, resulting in NMS. We conclude that combination therapy with carbamazepine and amitriptyline should be avoided in patients who are predisposed to NMS. The purpose of this paper is to warn physicians against combination therapy with carbamazepine and tricyclic antidepressants which may be conducive to neuroleptic malignant syndrome in susceptible patients.

## 1. Introduction

Neuroleptic malignant syndrome (NMS) was first reported by French psychiatrists in 1960 [1]. NMS is an uncommon but potentially fatal idiosyncratic reaction to neuroleptics characterized by muscular rigidity, fever, autonomic dysfunction, and altered consciousness. Pertinent laboratory findings include leukocytosis and an elevated serum creatine phosphokinase (CPK) level [1]. Although potent neuroleptics (e.g., haloperidol and fluphenazine) are more frequently associated with NMS, all antipsychotic agents, typical or atypical, may precipitate the syndrome. NMS has also been associated with nonneuroleptic agents that block central dopamine pathways (e.g., metoclopramide, amoxapine, and lithium) [2, 3]. NMS can present a clinical challenge where early diagnosis and intervention are essential to avoid a fatal outcome [4]. A molecular basis for NMS has not been established, however, studies suggest that genetic factors may play a part in the pathogenesis of NMS [5]. Involvement of serotonin pathways in NMS has been suggested, but disputed by the notion that polymorphisms in the 5-HT1A and 5-HT2A receptor genes do not determine susceptibility to NMS [6].

In this paper, we report, for the first time, the occurrence of NMS in a patient who received a combination of carbamazepine and amitriptyline.

## 2. Case Report

A 32-year-old Saudi female with a history of mental retardation and secondarily generalized convulsive seizures since childhood was admitted to King Khalid Hospital on December 19, 2011 because of altered mental status, poor feeding, and fever. For years, she had been treated with carbamazepine (CBZ) 200 mg TID with successful seizure control. Also, she had been followed by psychiatry for an unknown psychiatric illness, and was placed on amitriptyline 50 mg daily two months prior to admission.

 General physical examination showed a temperature of 39.8°C, a pulse rate of 88 beat/min, a blood pressure of 115/60 mmHg, and a respiratory rate of 22/min. Examination of the chest, cardiovascular system, and abdomen was unremarkable. There was no skin rash, or lymph node enlargement. The neurological examination showed the patient to be awake but confused. The pupils were 3 mm and reactive to light both directly and consensually. There was no papilledema, and extraocular muscle movements were intact. There was no facial asymmetry. She had generalized lead-pipe muscle rigidity with +2 deep tendon reflexes bilaterally. There were no Hoffmann's or Babinski's and no meningeal signs of irritation.

 On the first day of admission, a battery of tests was requested, and the results were as follows: CPK level was 692 U/L with an RBC of 3.35 million/*μ*L, Hb of 9.2 g/dL, and WBC of 3.97/cmm. Liver function tests, arterial blood gases, PT, PTT, INR, lipid profile, urine analysis, ESR, ECG, CXR, and CT scan of the head were all normal. MRI of the brain showed hypoplasia of the corpus callosum. A diagnosis of neuroleptic malignant syndrome (NMS) was made, and the patient was started on bromocriptine 2.5 mg tid. Carbamazepine and amitriptyline were both discontinued, and the patient was started on valproic acid for seizure therapy at a dosage of 250 mg bid.

On the 2nd day the CPK value rose to 1400 U/L, then to 2041 U/L on the same day. The temperature was 39.2°C. Cerebrospinal fluid studies were normal. At that time bromocriptine was increased to 5 mg TID, and IV fluid infusion rate was increased to 100 mL/hr. On the 3rd day of admission, a repeat CPK value was 3085 U/L, and the temperature was 38.5°C. Bromocriptine was increased to 7.5 mg TID. On the 4th day the patient became afebrile, and the CPK level dropped to 2696 U/L and later to 1358 U/L. On 28th of December (9 days after admission), the CPK level was 372 U/L. A few days later, her condition returned to baseline. Her therapeutic regimen at the time of discharge consisted of bromocriptine 5 mg TID and valproic acid 250 mg BID. She was given a follow-up appointment to the outpatient clinic in 2 weeks.

## 3. Discussion

 Our patient presented with a life-threatening condition manifested by autonomic dysfunction (hyperthermia and tachypnea), lead-pipe rigidity, and altered mental state. Her dramatic response to bromocriptine is in concert with previous reports of the efficacy of this drug against NMS [7].

 The incidence of NMS is about 1% in patients treated with antipsychotic medications. It is noteworthy that anticonvulsants and tricyclics (TCA) independently have been mentioned passingly as a potential cause of NMS, although solid data are lacking. Our data, for the first time, document objectively the occurrence of NMS during combination therapy with CBZ and TCA. Mortality is about 5–11.6%. Death usually occurs as a result of respiratory failure, disseminated intravascular coagulation, renal failure, and/or cardiovascular collapse [8].

Our patient had suffered from mental retardation, an unknown psychiatric illness, and epilepsy. She had been treated with CBZ since childhood for seizure control. Two months prior to admission she was also placed on amitriptyline.

 Carbamazepine (a tricyclic itself) is metabolized by Cytochrome3A4 [9], whereas tricyclic antidepressants (e.g., amitriptyline) are metabolized by Cytochrome450 [10]. While CBZ is known to reduce the serum level of amitriptyline when administered concurrently, there are no systematic data on the effect of amitriptyline on the serum level of CBZ [11]. However, there have been conflicting reports ranging from “no change” to “decreased” versus “increased” levels of CBZ when given in combination with TCA.

It is conceivable that in our patient, the addition of TCA to CBZ caused a decrease in the serum level of CBZ (a potentially dopaminergic drug), presumably through Cytochrome 3A4 induction, resulting in a state of relative “dopamine deficiency” which is known to be conducive to NMS.

 In conclusion, caution should be exercised when TCA is added to CBZ, particularly in patients in whom NMS risk factors exist (e.g., brain disease, dehydration).
